# Archaeal Viruses from High-Temperature Environments

**DOI:** 10.3390/genes9030128

**Published:** 2018-02-27

**Authors:** Jacob H. Munson-McGee, Jamie C. Snyder, Mark J. Young

**Affiliations:** 1 Department of Microbiology and Immunology, Montana State University, Bozeman, MT 59715, USA; jacob.munsonmcgee@msu.montana.edu; 2 Department of Biological Sciences, Cal Poly Pomona, Pomona, CA 91768, USA; jcsnyder@cpp.edu; 3 Department of Plant Sciences and Plant Pathology, Montana State University, Bozeman, MT 59715, USA

**Keywords:** archaeal virology, extremophiles, environmental virology, archaeal viral genetics, archaeal viral genes

## Abstract

Archaeal viruses are some of the most enigmatic viruses known, due to the small number that have been characterized to date. The number of known archaeal viruses lags behind known bacteriophages by over an order of magnitude. Despite this, the high levels of genetic and morphological diversity that archaeal viruses display has attracted researchers for over 45 years. Extreme natural environments, such as acidic hot springs, are almost exclusively populated by Archaea and their viruses, making these attractive environments for the discovery and characterization of new viruses. The archaeal viruses from these environments have provided insights into archaeal biology, gene function, and viral evolution. This review focuses on advances from over four decades of archaeal virology, with a particular focus on archaeal viruses from high temperature environments, the existing challenges in understanding archaeal virus gene function, and approaches being taken to overcome these limitations.

## 1. Introduction

Archaea are well known as extremophiles that can thrive in some of the most extreme and inhospitable environments [1]. Members of the Archaea appear to be an interesting blend of features typically found in bacteria (e.g., metabolic characteristics and overall cellular morphology) or in eukaryotic organisms (e.g., eukaryotic-like information processing systems) along with features that distinguish Archaea from both Bacteria and Eukarya (e.g., cellular membranes with ether linked lipids). The Archaea is comprised of several recognized phyla, including the better-studied Euryarchaeota and Crenarchaeota, and several candidate phyla, including the recently discovered superphylum Asgardarchaeota (Table 1) [2,3]. It is now appreciated that members of the Archaea can be found across diverse natural environments [4,5,6,7], however, their dominance in certain extreme environments makes these sites ideal settings for the discovery of archaeal viruses and for examining archaeal–virus interactions. Nearly all of the archaeal viruses that have been described to date have been isolated from either high-salt environments or high-temperature (>70 °C) hot springs, making them ideal tools with which to study the genes and genomics of biological entities from extreme environments. This review will focus on what we have learned from nearly 45 years of examining archaeal viral genes, and the significant challenges that remain in understanding the function of archaeal viral genes and their interactions with their hosts. This review will focus principally on studies of archaeal viruses from high temperature environments, but will also touch on archaeal viruses from other environments.

High-temperature low-pH hot springs around the world are dominated by Archaea to the point where no eukaryotic organisms are present, and Bacteria only make up a small portion, typically <1% of the cellular biomass in many hot springs [1,8,9,10]. One possible explanation for the dominance of Archaea in these environments is not that Bacteria are unable to tolerate and live in such extreme conditions, but rather, that Archaea are better at surviving in these conditions, due to their ability to outcompete Bacteria in environments where nutrients are chronically limited [11]. In addition to lacking bacterial species, these environments frequently have a low diversity of archaeal species present (typically <10) [8,12], replicating in a low cell density environment. These low-complexity cellular communities, combined with near complete knowledge of the viral community structure [13,14,15], make high-temperature low-pH hot springs ideal natural environments for the study of archaeal virus–host interactions in a natural setting.

**Figure 1:**
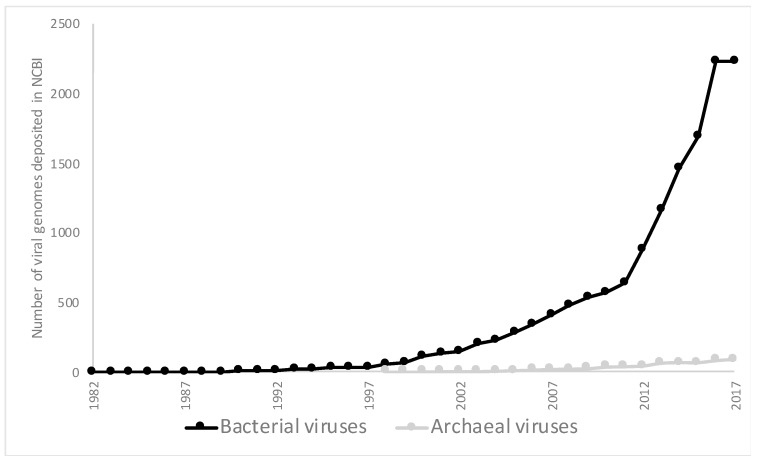
Number of bacterial and archaeal viruses that have been fully sequenced and deposited in the NCBI viral genome database on 6 December 2017 [27].

The number of viruses described from extreme environments has been increasing, but compared to mesophilic environments, the number of viruses described still lags significantly behind. The hot springs of Yellowstone National Park (YNP) and around the world have been especially rewarding for the study of thermophilic viruses infecting species of Crenarchaea. These viruses exhibit remarkable morphological diversity not shared by any other viruses that have been characterized to date, and are extensively reviewed by Dellas et al. [16], Rachel et al. [17], and Prangishvili et al. [18]. However, to date, only ~100 archaeal viruses, all of which have DNA genomes, as compared to over 2000 bacteriophages, with both DNA and RNA genomes, have been classified in the most recent release of the International Committee on Taxonomy of Viruses (ICTV) [19]. Of the archaeal viruses deposited in National Center for Biotechnology Information (NCBI) (Figure 1), most infect halophilic species within the Euryarchaeota phylum (32 viruses) and thermophilic species of Crenarchaeota (55 viruses). However, even when viruses that have only been identified through metagenomic approaches are included, there are still only roughly 150 archaeal viruses that have been described, to date. In addition, the viruses infecting members of most archaeal phyla are unknown at this time (Table 1). 

## 2. Challenges Associated with Archaeal Virology

One reason for the relatively small number of archaeal viruses that have been described is due to the difficulty of culturing many of their hosts. Metagenomic approaches have helped to overcome this limitation, and have increased our knowledge of viruses from extreme environments. One case study of a single hot spring from YNP revealed 110 viral groups [13], only seven of which have characterized representatives. Interestingly, this same hot spring has <10 host species [8], suggesting that we have only begun to scratch the surface of the diversity of extreme viruses present within these environments.

Despite the small number of archaeal viruses that have been characterized to date, the viruses of Archaea are classified into over 17 families, reflecting the wide morphological and genetic diversity of these viruses [28]. In comparison, the viruses infecting Bacteria are classified into only 10 families, despite there being over 2000 fully sequenced bacteriophages in the NCBI viral database [27]. The genomes of archaeal viruses exhibit remarkable genetic diversity from each other and from bacteriophages. A frustration (and source of excitement) for researchers studying novel archaeal viruses is the tendency for 75–90% of genes in a new archaeal virus to have no significant match (BLAST <1 × 10^−5^) in the public databases, making the identification of gene function difficult. While bacteriophages have a similar problem, it is not as dramatic as in crenarchaeal viruses, where the function of ~85% of viral genes are unknown [28]. However, when crenarchaeal viral gene functions have been determined, they have often led to major new insights into archaeal biology. For example, a completely new type of virus release mechanism was identified based on the formation of seven-sided pyramid-like structures on the surface of infected cells [29,30]. Overall, there are many functions encoded by crenarchaeal viruses that are akin to functions seen in eukaryotic viruses, including interactions with the cellular endosomal sorting complex required for transport (ESCRT) system [31,32], RNA polymerase [33], and similarity in rolling circle replication proteins [34,35]. Additionally, the structures of the *Sulfolobus* turreted icosahedral virus (STIV) and *Haloarcula sinaiiensis* tailed virus 1 (HSTV-1) (Table 2) major capsid proteins were found to be homologous to major capsid proteins of viruses infecting Bacteria and eukaryotes, suggesting an ancient origin of these viral lineages [36,37]. There is also a blurring of the lines between archaeal viruses and archaeal plasmids. A recent study described a plasmid from an Antarctic archaeon that is transported between cells by a vesicle that also has plasmid-encoded proteins embedded in the lipid membrane [38]. While this plasmid has no obvious viral hallmark genes, it is worth noting that neither do pleolipoviruses [39], which these vesicles resemble morphologically, and also infect haloarchaea [40].

The lack of understanding of genes from thermophilic viruses is exemplified by prokaryotic viral orthologous group (pVOG) analysis [54]. Orthologous protein clustering has become a widely accepted method to computationally identify protein orthologs, with the added benefit that the protein function does not need to be known for orthologs to be identified. Protein clustering approaches have been applied to viral systems from the open ocean [55,56], to virophages that co-infect eukaryotic viruses with a giant virus [57], and to undescribed viral “dark matter” [58]. 

**Figure 2:**
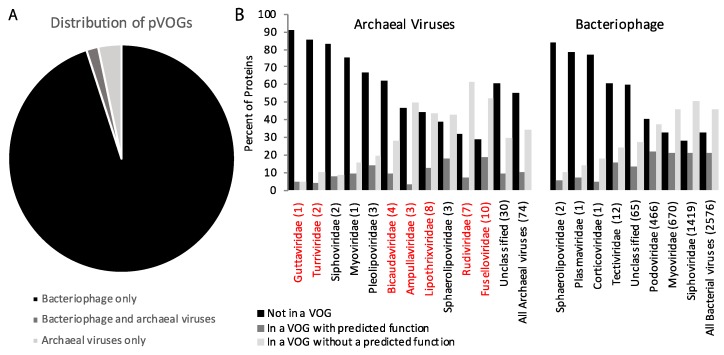
Analysis of genes from double stranded DNA (dsDNA) viruses in the prokaryotic viral orthologous group (pVOG) analysis [54]. (**A**) Distribution of pVOGs that are encoded for by dsDNA bacteriophages only, dsDNA archaeal viruses only, or both. (**B**) Percentage of genes from each viral family for dsDNA archaeal viruses and bacteriophages that are not in a VOG, in a VOG with a predicted function, or in a VOG that lacks a predicted function. For each viral family, the number of viruses belonging to the family is provided in parenthesis. Archaeal viral families with high temperature representatives are shown in red.

Recently, all genes encoded by archaeal and bacterial viruses were clustered into nearly 10,000 pVOGs [54]. While there were nearly 3000 bacteriophages included in this analysis, there were only 78 archaeal viruses. Of the archaeal viruses, 74 had double stranded DNA (dsDNA) genomes. Due to the absence of archaeal RNA viruses, and the small number of archaeal single stranded DNA (ssDNA) viruses, RNA, and ssDNA bacteriophages, these were omitted from our analysis. Of the genes encoded by dsDNA bacteriophages, 65% clustered into one of the pVOG. However, for dsDNA archaeal viruses, only 43% of genes were part of a pVOG (Figure 2). A further analysis reveals that genes of archaeal viruses are more likely to not be part of a pVOG or part of a pVOG where the function is unknown (90%) than the genes of bacterial viruses (79%). In fact, for many archaeal viral families, genes are primarily not part of a pVOG or are a member of a pVOG with an unknown function (Fig 2). Overall, these observations indicate that archaeal virus gene annotation lags behind bacteriophage gene annotation.

## 3. Comparison of Gene Content of Bacteriophage, Archaeal, and Eukaryotic Viruses

Despite the overrepresentation of bacteriophage in the pVOG dataset, there are a number of pVOGs that are only encoded by archaeal viruses. Of the 479 pVOGs that are encoded for by dsDNA archaeal viruses, 66% (317 pVOGs) are encoded only by archaeal viruses without membership from any bacteriophage. As expected, the vast majority of these archaeal-only pVOGs do not have a predicted function. When combined with the archaeal virus genes that are not part of a pVOG, only ~10% of all archaeal dsDNA viral genes are part of a pVOG with a predicted function. These results indicate that a significant component of the archaeal virus gene repertoire differs from their bacteriophage counterparts. 

The genomes of archaeal viruses differ from bacterial viruses at the whole genome level as well. The known archaeal viruses, on average, have smaller genomes than bacteriophages [28] and consequently encode for fewer proteins (65 open reading frames (ORFs) on average for archaeal viruses as compared to 101 ORFs for bacteriophage). Archaeal viruses also encode for significantly more genes per kb than bacteriophage (1.62 to 1.49 genes/kb respectively, *p* < 0.01, *t*-test). Packaging of the archaeal viral genomes within their virion appears to involve more diverse mechanisms. For example, *Sulfolobus islandicus* rod-shaped virus 2 (SIRV2) packages its genome as A-form DNA [59]. While A-form DNA had previously been described in bacterial spores [60], this was the first time that it had been observed in a virus. A-form DNA is found in multiple biological entities exposed to harsh conditions, suggesting that packaging DNA in this form may be a common mechanism for the protection of DNA in the most inhospitable environments. Likewise, the virus *Aeropyrum pernix* bacilliform virus 1 (APBV1) tightly packages its DNA genome as a left-handed superhelix with its major capsid protein [61]. Both of these examples likely reflect adaptations for DNA stability at high temperatures.

Most high-temperature low-pH hot springs present numerous challenges that must be overcome by viruses and their hosts. As a result of being low-nutrient environments in combination with high-metal and or salt concentrations, most high-temperature low-pH hot springs have cell densities lower than seawater. This low host abundance makes it difficult for viruses to find a new host after release through lysis [62] or budding [63] from an infected cell. The difficulty of finding a new host is further complicated by the fact that the half-life of archaeal viruses at high temperatures is often short (t_1/2_ <60 min) (unpublished data [64]). To overcome this challenge, some archaeal viruses have developed novel mechanisms to increase their ability to find host cells. One example of this is the *Acidianus* two-tailed virus (ATV), which, upon release from the host cell, undergoes a dramatic conformational change, where the central spindle shape of the virion contracts in width, while the two tails extend in length up to 1 μm [65]. This morphological change occurs in the absence of the host cell, energy source, or external cofactors [65]. Such a dramatic morphological change is hypothesized to assist the virus in its search for a new host, by dramatically increasing the area it can search and by minimizing the time that the viral particle spends unprotected outside of a host cell. 

The difficulty of finding a new host also suggests that the viruses of thermophilic Archaea might have a greater tendency toward vertical vs horizontal transmission mechanisms within their environments. Among cultured thermophilic archaeal viruses, almost all are capable of establishing chronic or persistent infections, and only a small number, among them ATV and *Thermoproteus tenax* virus 1 (TTV-1), are obligately virulent [66]. Interestingly, halophilic viruses seem to be more likely to be lytic [67], although in some halophilic viruses, integrases have been identified [68], and others seem to be capable of persistent infections [69]. This suggests that the conversion to lysogeny is possibly an adaptation to counter the harsh environment encountered by thermophilic and halophilic viruses outside of their host cells. This ability to propagate vertically while still generating a small number of progeny would be extremely beneficial in an environment where finding a host cell and successfully establishing an infection is probably one of the greatest limitations. The two factors discussed above (low cell density and short virus half-life in the environment) both promote a viral lifestyle that minimizes the amount of time that a virus spends outside of its host cell. As a result, these viruses are more likely to replicate via a chronic or lysogenic lifecycle. 

While the complete lifecycle of most archaeal viruses remains unknown, there are several steps of the replication cycle that are beginning to be understood. Many of these processes have remarkable similarity to eukaryotic viral processes that are not as commonly observed in bacteriophage replication cycles. Archaeal viruses of the *Rudiviridae* family have covalently closed linear dsDNA genomes similar to members of the eukaryotic viral family *Poxviridae*. The similarity of these genome structures led to the proposal of the flip-flop model of genome replication for SIRV1 [35]. A new archaeal virus, *Metallospheara* turreted icosahedral virus (MTIV), has a linear dsDNA genome with inverted terminal repeats (ITRs) [47] that are likely involved in genome replication, like adenovirus [70], and some herpesviruses [71] and bacteriophages of the family *Tectiviridae* [72]. It is still to be determined if MTIV utilizes a protein to prime genome replication, like adenovirus [47], or if it is self-primed, like ssDNA eukaryotic viruses such as human parvovirus [73]. Many other archaeal viruses contain ITRs, including *Methanosarcina* spherical virus (MetSV), which likely replicates via protein-primed DNA replication [46]. 

Common to archaeal and eukaryotic viruses is the discovery that the archaeal homolog of the endosomal sorting complex required for transport (ESCRT) is utilized by some archaeal viruses for viral assembly and escape [31,32]. This same complex is also utilized by several eukaryotic viruses, including human immunodeficiency virus (HIV), to facilitate egress from their host cell [32]. Archaeal viruses budding from their host cells appears similar to the budding process utilized by many enveloped eukaryotic viruses [63]. However, these processes are not commonly found in bacteriophages. It will be interesting if additional viral processes are discovered that are shared between archaeal and eukaryotic viruses, supporting a hypothesis that like their cellular counterparts, archaeal and eukaryotic viruses are more similar to each other than they are to bacteriophages. The discovery and characterization of viruses infecting the recently described archaeal superphylum Asgardarchaeota [3], which forms a monophyletic clade with Eukarya, will likely shed insights into the evolutionary relationships between viruses infecting the Archaea and eukaryotes. 

The shared cellular architectures and information processing pathways between Archaea and eukaryotes provide commonalities between these domains that viruses can exploit to replicate. These commonalities also mean that the study of archaeal viruses can be used to shed light on processes in eukaryotic cells and vice versa. In a recent example, cellular *TFS4*, which acts as potent inhibitor of host RNA polymerase, was found to be induced upon infection of a *Sulfolobus* species by STIV [33]. A chimeric version of this protein is able to inhibit RNA polymerase activity in yeast cells, suggesting that this method of RNA inhibition is conserved between Archaea and Eukarya [33]. While it is unknown if this protein maintains its function in other eukaryotic systems, this discovery does raise the speculation that viruses exploit other mechanisms conserved across domains. 

## 4. Archaeal Virus Life Style and Gene Functions

Many archaeal viruses, as well as some bacteriophages, utilize pili and flagella in their initial binding to host cells [74]. While there are no archaeal viruses for which the cellular receptor has been identified, there are numerous descriptions of archaeal viruses attaching to extracellular structures, most notably pili. These include STIV [64] and *Acidianus filamentous* virus 1 (AFV1), which attaches to the cell pili with claw-like structures on the distal part of the virion [41]. SIRV2 also attaches to the tips of pili, and then proceeds to move along the pili towards the cell [75]. This binding occurs within minutes after addition of virus in a cultured setting [75], and supports the hypothesis that thermophilic viruses have evolved to minimize time spent outside of the cell. 

Many archaeal viruses may establish chronic infections within their host cells. As such, the virus may spend the majority of its life cycle within the infected cell. The fact that ~90% of Archaea have CRISPR (Clustered Regularly Interspaced Short Palindromic Repeats)/Cas (CRISPR Associated) anti-virus defense system [76] suggests that archaeal viruses have effective mechanisms to overcome CRISPR/Cas systems. A recent study found that upon infection with a single virus CRISPR, spacer acquisition did not happen. However, upon infection with a second virus, hyperactive CRISPR spacer acquisition was observed from one virus but not the second virus, indicating that some archaeal viruses have evolved anti-CRISPR mechanisms to protect their DNA from spacer acquisition [77]. CRISPR loci have also been detected in some archaeal viral genomes, although their purpose is not yet known [43]. However, it tempting to speculate that they may function as a virus-based anti-viral based system to exclude competing viruses from infecting the same cell. The identification of specific proteins that are involved in countering archaeal CRISPR systems, and characterization of their function, will remain an area of interest in the years to come.

A major challenge moving archaeal virology forward is how to elucidate the function(s) of archaeal virus genes and their interactions with their host machinery. An enhanced effort on the biochemical analysis of archaeal host and viral genes will undoubtedly prove worthwhile. For example, two proteins within the genomes of SIRV1 and SIRV2 appeared to be homologous to Holliday junction resolving enzymes [78]. While each gene showed an 85% identity to each other, they had very limited homology to other Holliday junction resolvases (34% homology in the N-terminal half and only 6% in the C-terminal half). Heterologous expression and purification of the SIRV proteins showed preferential binding to cruciform DNA at temperatures up to 70 °C. Furthermore, these proteins were shown in vitro to be capable of resolving replication intermediates, genetic recombination, and DNA repair.

Combining genetic and biochemical analysis has proven a powerful approach to determine gene function. The development of archaeal host and viral genetic systems is limited, but where it has been accomplished, it has proven to be quite useful. While most Crenarchaea remain genetically intractable, significant advances have been made in the creation of a suite of tools for the genetic manipulation of *Sulfolobus* species. These tools include viral (SSV1) and plasmid-based shuttle systems, as well as CRISPR based gene deletion, mutagenesis, and silencing techniques [79]. Genetic systems have also been developed for some archaeal viruses, for example, the SSV1 genetic system has provided insightful into the function of archaeal viral genes [80,81,82]. Both random and targeted mutagenesis of the SSV1 viral genome surprisingly revealed that only half of the viral genes encoded by the SSV1 genome (16/35 viral genes) are essential for infectivity [81]. Furthermore, the requirement for a particular viral gene correlates well with its degree of conservation among the *Fuselloviridae*. 

In a second example, combined genetic and biochemical analysis of *c92* gene from STIV1 revealed its function in a new cell lysis mechanism [30,62], and metagenomic analysis revealed that it is likely widespread in archaeal viruses of acidic hot springs [83]. A final example: combined genetics and biochemical assays to suggest the function of viral gene, *ORF79*, from the halophilic virus ϕCh1. Bioinformatically, ORF79 shows low homology to another halophilic viral protein, gp5 from *Haloarcula hispanica* tailed virus 2 (HHTV-2), to the adenovirus E1A protein, and to chromatin remodeling proteins [84]. Transformation of a ORF79 disruption cassette into a strain of the host that carried a proviral ϕCh1 yielded viruses that carried a disrupted version of ORF79. The mutant strains showed a premature onset of viral lysis in comparison to wild type virus. Furthermore, expression of ORF79 in a lysogenic strain of *N. magadii* resulted in inhibition of lysis.

Structural studies often provide valuable insights into unknown archaeal virus protein function(s). One example of this is the structural analysis of major capsid protein of STIV. This protein lacks significant similarity to other proteins in public databases but, based on secondary structure analysis, was predicted to have the “double jelly roll” motif found in viruses infecting Bacteria and eukaryotes [37]. Combined structural analysis using X-ray crystallography and cryo-electron microscopy showed remarkable similarity of STIV major coat protein to the capsid proteins of PRD1 and *Paramecium bursaria* chlorella virus 1 (PBCV-1), suggesting an ancient lineage for a group of viruses infecting all three domains of cellular life [37]. A decade later, the archaeal virus HSTV-1 was shown to have a HK97-like protein fold found in some bacteriophage and eukaryotic viruses, providing evidence of two ancient viral lineages that existed prior to the split of Archaea and Bacteria [36]. 

One of the earliest structural studies of archaeal viral proteins focused on the STIV A197 protein [85]. While the sequence of this viral protein provided little insight into its function, the structure revealed that it belonged to the glycosyltransferase superfamily. These proteins have large diversity, which can make identifying them by sequence similarity challenging. A197 from STIV belongs to the GT-A superfamily of glycosyltransferases that is hypothesized to have evolved from a common ancestor [86]. Glycosyltransferases are ubiquitous to the three domains of life, indicating they were present prior to the separation of the domains of life. In recent years, several other proteins from STIV have had their structure determined, providing insights into their function: B204 is an ATPase, and likely functions in DNA packaging and/or release from the virion [87]; F93 [88] and B116 [89] are two DNA binding proteins likely involved in regulation of host and/or viral transcription; A81 forms a proliferating cell nuclear antigen (PCNA)-like ring structure likely involved in DNA replication [90]; and A223 and C381 proteins form the turrets extending from the surface of STIV virions, which are likely involved with genome packaging and release, as well as binding to the uninfected cell surface [91].

More recently, tomography and single particle cryo-electron microscopy have been applied to structural studies of virions and host–virion interactions. Cryo-tomography has been used to study the interaction of the STIV turrets with fibers extending from the host cell surface (unpublished data [64]). The large spindle-shaped archaeal virus, *Acidianus* tailed spindle virus (ATSV), was recently examined by tomography and, unlike bacteriophages with distinct head and tail structures, the head and tail of ATSV appear to be a single continuous structure [92], suggesting a new virion assembly mechanism [93]. The capsid structure of a thermophilic virus MTIV was solved to 22Å using a combination of tomography and single particle reconstruction, revealing a new virion architecture [47]. Additionally, cryo-electron microscopy has been used to study the hyperthermophilic virus SIRV2 virion, to discover that almost half of the major capsid protein remains unstructured, and the viral DNA of SIRV2 is entirely packaged in A-form [59]. 

In recent years, culture-independent approaches have been used to identify virus–host interactions. This has been accomplished through a number of techniques, including viral tagging [94,95], phage fluorescent in situ hybridization (FISH) [43,47,96], single cell genomics (SCG) [97,98], and bioinformatic analysis of previously published sequence data [58,99,100]. While these techniques have produced limited insight into the structure and function of viral genes, they have frequently aided researchers in identifying the appropriate host to direct culturing efforts [43]. While difficult, culture-independent studies can provide insight into viral genes in combination with other techniques. For example, the large-tailed spindle virus ATSV was initially isolated directly from environmental hot spring samples, and its host identified using both viral FISH and cellular CRISPR/Cas analysis [43]. Prior to host identification, the major coat protein was identified, cloned, and the structure was solved [92], which lead to hypothesis about viral evolution, gene conservation, and novel mechanisms for viral [93]. One promising approach is the application of SCG to environmental samples. SCG has the ability to directly identify virus–host interactions, superinfections, and to help define viral host ranges. Further advances in the technology will allow for the detection of viral RNA, to identify replicating viruses within single cells. FISH also has the ability to study viruses interacting with their hosts in environmental settings. Phage FISH is capable of identifying novel virus–host relationships [96], while gene FISH is able to detect specific viral genes of interest [101,102] throughout the virus infection.

Despite their ubiquity (all viruses have at least one), structural proteins remain difficult to identify in novel thermophilic viruses. The low level of sequence conservation, and multiple possible protein folds, makes bioinformatic identification of these proteins difficult. While the genes of these proteins are the closest there is to a universal viral gene marker, the unknown degree of transfer between viral families makes a definitive phylogenetic analysis based on viral structural protein alone, difficult. Environmental viral metaproteomics, in combination with protein clustering techniques, is one of the most promising high-throughput techniques for the identification and characterization of viral proteins. For example, a single environmental viral metaproteomic study identified 1875 virion-associated open-ocean proteins which are widespread and abundant [103]. Four of the five most abundant proteins contained the HK97-like fold that is found in viruses that infect all three domains of life [36]. Combining the dataset with protein clustering assigned a functional annotation to 47 of the most abundant protein clusters [103]. However, viral metaproteomics is limited in that viral proteins that are not associated with the viral capsid will not be detected and characterized. 

## 5. Future Prospects

Looking forward, there is still a vital need for discovery and characterization of archaeal viruses from other environments, including, but not limited to, deep-sea vents, where certain lineages of Asgardarchaeota were identified [2], and additional hot springs, marine systems, and other environments where Archaea are prevalent [104]. To date, only two phyla of Archaea have cultured viruses (Crenarchaeota and Euryarchaeota), and several others have metagenomic evidence of viruses (Table 1). Additionally, the further refinement of bioinformatic tools to match viruses to their hosts based on CRISPR spacer matches, k-mer usage frequency, or other techniques, will provide invaluable avenues for researchers to study archaeal viruses in other environments where Archaea are present in lower frequencies. These studies will discover new archaeal viral families, as well as describe new members of existing archaeal viral families, allowing for a more in-depth examination of the evolution of archaeal viruses and their hosts. While laboratory studies offer invaluable information and insight as to how viruses interact with their hosts, they do not tell the whole story. There is a need to develop additional tools to probe host–virus interactions dPleairectly in their environments. It is likely that many of the unknown viral gene products function only in the context of their environment, helping to overcome host defense systems, outcompeting other viruses, and adapting to changes in the geochemical environment that is often found in extreme environments. 

While the field of archaeal virology has advanced in recent years, there still remains significant challenges in understanding archaeal viral gene function. The expanded appreciation that Archaea exist well beyond extreme environments challenges us to understand their role in the ecology, and evolution of organisms within these environments. This will only occur by more fully understanding archaeal viral gene function and interactions with their hosts, both in the laboratory and in their natural environments. The fascinating gene diversity exhibited by these viruses should attract increased attention from virologists, biochemists, genetics, and structural biologists to unravel archaeal virus function. Future advances in culturing and the development of genetic systems will undoubtedly provide excellent systems for the study of archaeal viruses. 

## Figures and Tables

**Table 1:** Number of isolated viruses infecting archaeal phyla and candidate phyla with metagenomic evidence of viruses. Archaeal phyla and candidate phyla were curated from the National Center for Biotechnology Information (NCBI) taxonomy browser [20] on 9 February 2018. The number of viruses was determined from the NCBI viral database. Numbers in brackets refer to articles that describe viruses from metagenomic sequences that are inferred to infect the archaeal phyla.

Phyla	Number of Viruses in NCBI	Metagenomic Viruses
Candiditus Aenigmarchaeota	0	No
Candiditus Bathyarchaeota	0	Yes [21]
Crenarchaeota	55	Yes [13]
Candiditus Diapherotrites	0	No
Euryarchaeota	32	Yes [21,22,23]
Candiditus Geothermarchaeota	0	No
Candiditus Heimdallarchaeota	0	No
Candiditus Korarchaeota	0	No
Candiditus Lokiarchaeota	0	No
Candiditus Micrarchaeota	0	No
Nanoarchaeota	0	Yes [8]
Candiditus Nanohaloarchaeota	0	Yes [24]
Candiditus Odinarchaeota	0	No
Candiditus Pacearchaeota	0	No
Candiditus Parvarchaeota	0	No
Thaumarchaeota	0	Yes [23,25,26]
Candiditus Thorarchaeota	0	No
Candiditus Woesearchaeota	0	No

**Table 2:** Table of all viruses mentioned in this review, their abbreviation, and the original reference where the virus is described.

Abbreviation	Virus Name	Reference
AFV1	*Acidianus* filamentous virus 1	Bettstetter et al., 2003 [41]
APBV1	*Aeropyrum pernix* bacilliform virus 1	Mochizuki et al., 2010 [42]
ATSV	*Acidianus* tailed spindle virus	Hochstein et al., 2015 [43]
ATV	*Acidianus* two-tailed virus	Prangishvili et al., 2006 [44]
HHTV-2	*Haloarcula hispanica* tailed virus 2	Atanasova et al., 2012 [45]
HSTV-1	*Haloarcula sinaiiensis* tailed virus 1	Atanasova et al., 2012 [45]
MetSV	*Methanosarcina* spherical virus	Weidenbach et al., [46]
MTIV	*Metallosphaera* turreted icosahedral virus	Wagner et al., 2017 [47]
ϕCh1	*Natrialba magadii* phi Ch1	Witte et al., 1997 [48]
PBCV-1	*Paramecium bursaria* chlorella virus 1	Reisser et al., 1988 [49]
PRD1	Phage PRD1	Olsen et al., 1974 [50]
SIRV1	*Sulfolobus islandicus* rod-shaped virus 1	Prangishvili et al., 1999 [51]
SIRV2	*Sulfolobus islandicus* rod-shaped virus 2	Prangishvili et al., 1999 [51]
SSV1	*Sulfolobus* spindle virus 1	Palm et al., 1991 [52]
STIV	*Sulfolobus* turreted icosahedral virus	Rice et al., 2004 [37]
TTV-1	*Thermoproteus tenax* virus 1	Janekovic et al., 1983 [53]
